# The regulation of ovary and conceptus on the uterine natural killer cells during early pregnancy

**DOI:** 10.1186/s12958-017-0290-1

**Published:** 2017-09-06

**Authors:** Han Gong, Yilu Chen, Jingjie Xu, Xingxing Xie, Dainan Yu, Bei Yang, Haibin Kuang

**Affiliations:** 1 Department of Physiology and Jiangxi Provincial Key Laboratory of Reproductive Physiology and Pathology, Basic Medical College, Nanchang University, Nanchang, Jiangxi 330006 People’s Republic of China; 2 Department of Clinic medicine, School of Queen Mary, Nanchang University, Nanchang, Jiangxi 330006 People’s Republic of China; 3 Department of Physiology, Basic Medical College, Nanchang University, Nanchang, Jiangxi 330006 People’s Republic of China

**Keywords:** Uterine natural killer, Ovary, Conceptus, Regulation, Pregnancy

## Abstract

Uterine natural killer (uNK) cells are short-lived, terminally differentiated and the most abundant lymphocytes in the uterus which play a crucial role in the spiral arteriole modification and establishment of successful pregnancy. Dysregulation of uNK cells has been linked to gestational implications such as recurrent pregnancy loss, preeclampsia and fetal growth retardation. There is evidence showing that progesterone and estrogen can regulate the recruitment, proliferation, differentiation and function of uNK cells via direct action on intracellular nuclear receptors or through intermediary cells in the uterus during early pregnancy. As the deepening of related research in this field, the role of conceptus in such regulation has received extensive attention, it utilizes endocrine signaling (hCG), juxtacrine signaling (HLA-C, HLA-E, HLA-G) and paracrine signaling (cytokines) to facilitate the activities of uNK cells. In addition, under the influence of ovarian hormones, conceptus can increase expression of PIBF and HLA-G molecules to reduce cytotoxicity of uNK cells and promote angiogenesis. In this review, we aim to concentrate on the novel findings of ovarian hormones in the regulation of uNK cells, emphasize the regulatory role of conceptus on uNK cells and highlight the proposed issues for future research in the field.

## Background

Uterine natural killer (uNK) cells are short-lived, terminally differentiated and the most abundant granulated lymphocytes present in the non-pregnant endometrium and pregnant decidua of human uteri [1, 2]. In non-pregnant endometrium, the proportion of uNK cells in the endometrial stromal cells increases since the proliferative phase (10%) of menstrual cycle and reaches the maximal level in the late secretory phase (20%). After pregnancy, the proportion sustains to increase due to a large influx of NK lymphocytes from peripheral circulation (30%) [2] and the cells differentiate to present abundant cytoplasmic and membrane-bound granules and enlarge to 50 mm in diameter. Uterine NK cells are transient and begin to apoptosis to a much less prominent population of lymphocytes after early pregnancy [3]. Immunophenotyping experiments in both rodents and humans indicate that these pregnancy-associated transient lymphocytes resemble the CD56bright circulatory NK cell (cNK) subsets [4]. These cells are phenotypically identical to the typical NK cells, which are characterized as presence of CD56^+^ and CD3^−^. In addition, they also lack of CD16, an important mediator of antibody-dependent cellular cytotoxicity (ADCC) for NK cells to lyse target cells and are less cytotoxic than other subsets of cNK cells. The ability to produce large amounts of cytokines upon activation is another important characteristic for these cells [5]. Especially for uNK cells, which are shown to play an important role in early pregnancy, secreting cytokines is the main strategy to regulate trophoblast invasion, spiral arterial modification, placental formation and finally establish successful pregnancy [6].

Uterine NK cells also have additional characteristics that are unique to themselves [1, 7]. A recent microarray analysis has provided a detailed comparison of gene expression between uterine NK cells and their corresponding CD56bright NK population present in circulatory blood vessels [8]. The significant differences include selective overexpression of lectinlike receptors (NKG2C, NKG2E), KIRs and other potential immunoregulatory proteins (Galetin-1 and Glycodelin) in uNK cells but not in cNK cells. One possible explanation for the observed differences refers to uNK cells represent a distinct lineage of NK cells from hematopoietic precursors. Otherwise, the distinctions are probably a direct reflection of CD56bright NK cells differentiation in the uterine microenvironment. Previous reviews have summarized the role of estrogen and progesterone in the regulation of uNK cell recruitment, proliferation, differentiation and function via direct action on intracellular nuclear receptors or through intermediary cells in the uterus during early pregnancy [9, 10]. In this review, we emphasize the regulatory role of conceptus that have not been described before and are critically dedicated to construct a thorough regulatory network of uNK cells during early pregnancy.

### Regulation of ovary on the uNK cells

The anterior pituitary gland starts to synthesis follicle stimulating hormone (FSH) and luteinizing hormone (LH) since puberty and stimulate ovarian cells to synthesis progesterone and estrogen in a cyclic manner. After ovulation, the levels of progesterone and estrogen reach a peak to create a “window of implantation” 6–10 days and do not fall until the end of 10th week of gestation in human. After that, conceptus-derived placenta replaces ovarian cells to secret progesterone and estrogen.

### Regulation of estrogen and progesterone on the proliferation and recruitment of uNK cells

The changes that occur in uNK cell number in early pregnancy are attributed to self-renewal or trafficking of cNK cells [6]. Regulatory evidence of estrogen in uNK self-renewal is not entirely clear. Administration of estrogen in the culture medium did not significantly affect the proliferation of uNK cell in vitro [11]. However, in tamoxifen (anti-estrogen)-exposed mouse, proliferative activities of uNK cell were observed to be interfered in vivo [12], which may be account of permissive role of progesterone in vivo or other factors. However, mechanism of progesterone-mediated self-renewal is much clear. Some studies have showed that progesterone can stimulate endometrial stromal cells secreting IL-15 to promote self-renewal of uNK cells [13].

Both estrogen and progesterone play an indispensable role in uNK cell recruitment. Progesterone was initially found to upregulate VEGF and VEGF receptors on the endometrial stromal cells in an in vitro model of decidualization. VEGFs are factors required in angiogenesis, so they can improve histological perfusion to assist uNK cells homing [14]. Subsequent experiment has showed that both estrogen and progesterone can also increase homing through the increased expression of L-selectin and α-integrin on the surface of circulatory CD56bright NK cells [15] and CXCL10/CXCL11 on endometrial cells [16]. Recently, a study carried out by our lab has provided more visualized evidence to their recruited action. Uterine NK cells did not appear in mice uterus on day 2 of pregnancy, a time with low or no estrogen and progesterone. However, they began to distribute in uterine blood vessels in next day 3 and 4 of pregnancy and the distributing pattern of them is identical to the ovariectomized mice after administration of estrogen and progesterone, which further confirmed their role in homing of uNK cells during early pregnancy [17].

### Regulation of estrogen and progesterone on the function of uNK cells

Estrogen and progesterone can regulate the function of uNK cells through a direct or an indirect way. In direct way, estrogen and progesterone couple to their nuclear receptors to activate gene expression of immunomodulatory or angiogenic proteins in the uNK cells. In mice model, they have been shown to upregulate the expression of galectin-1, an immunosuppressant, in decidual NK population [9]. However, there is a slight difference in their regulatory effect on angiogenesis of uNK cells. Estrogen increases secretion of CCL2 in uNK cells to construct blood vessels in endometrium [18], while progesterone induces the expression of IFN-γ [19]. In indirect way, endometrial stromal cells, trophoblast cell, T lymphocyte are proposed intermediary cells transducing effects of progesterone. In response to progesterone, the cells produce Hoxa-10 [20], progesterone-induced blocking factor [21], and Th2 cytokines [22] respectively to reduce cytotoxicity of uNK cells. In general, both direct and indirect ways are necessary for maintenance of pregnancy.

### Potential mechanisms of steroid hormones action on the uNK cell

**Fig. 1:**
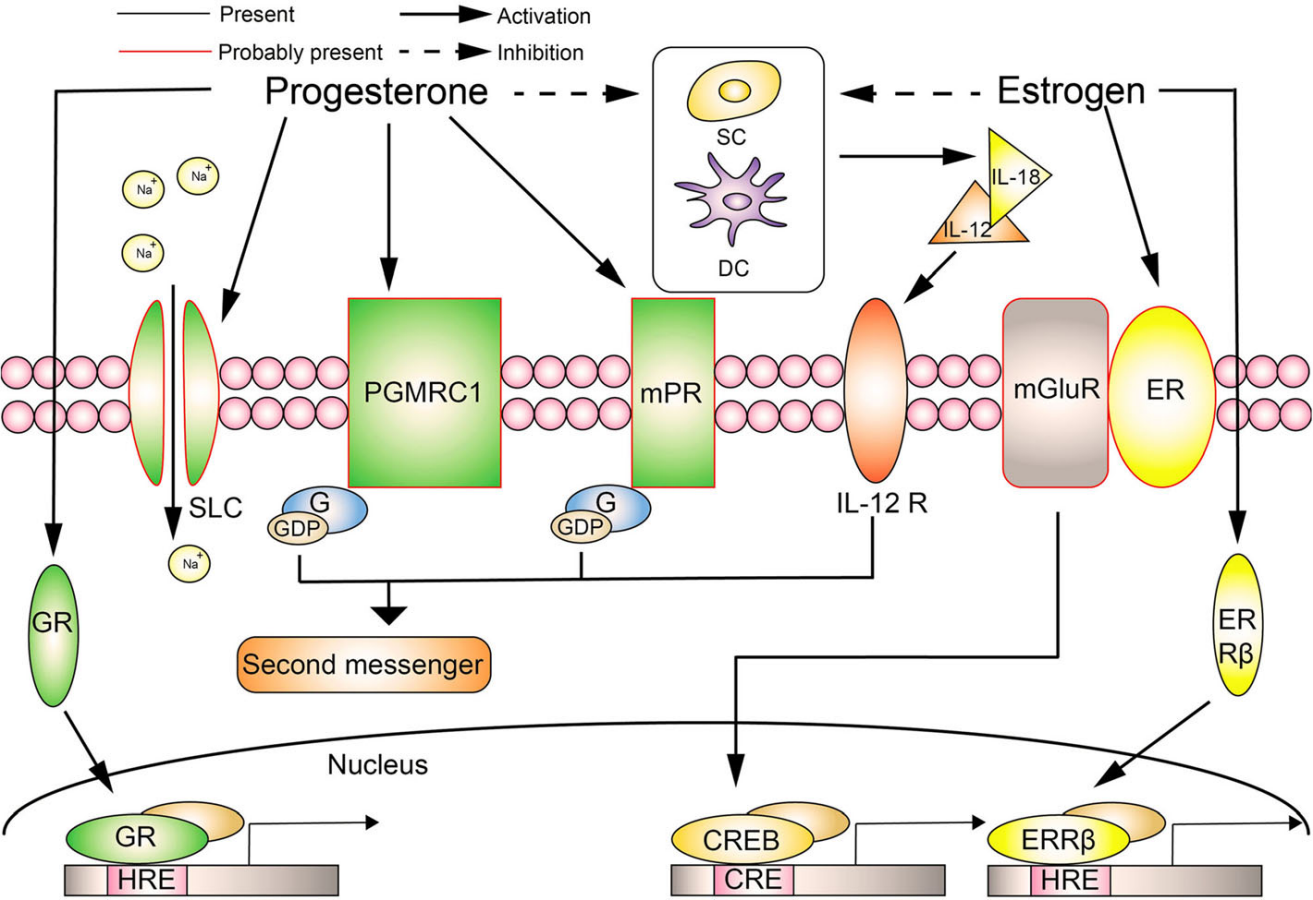
Potential mechanisms of steroid hormones action on the uNK cells. Through classical steroid receptors (e.g. GR, ERRβ), non-classical pathways or intermediary cells in the surrounding, progesterone and estrogen probably affect gene transcription, secondary messenger and membrane potential to regulate the activities of uNK cells. Among them, non-classical pathways referring to ion channels (e.g. SLC) and membrane-bound receptors are mainly contributed to rapid actions of the hormones. For progesterone, PGMRC1 and mPR are two potential candidates of membrane-bound receptors on the uNK cells and function as G protein-coupled receptors to activate or inhibit downstream G protein. For estrogen, its membrane-bound receptors may be coupled to mGluR2 and initiate intracellular signaling pathway of mGluR2 to regulate uNK cells activities. Otherwise, both progesterone and estrogen are likely to reduce IL-18 level in endometrial stromal cell (SC) and dendritical cells (DC) to inhibit cytotoxicity of uNK cells and improve pregnant outcomes

Biological activities of steroid hormones are predominantly mediated by binding to respective nuclear receptor to initiate gene transcription, which is considered as the most classical mechanism of steroid hormones. In 1996, Henderson et al. confirmed mRNA expression of ERβ1, ERβcx/β2 and glucocorticoid receptor (GR) in purified uNK cells, but failed to find ERα or progesterone receptor (PR), and further colocalized immunohistochemistry technique performed on uNK cells only confirmed presence of protein ERβ1 and GR [23]. However, when ERα and ERβ knock-out bone marrows were transplanted in RAG-2−/−/γc−/− mice, a mutant lack of all lymphocyte lineages, uNK cells were presented in a cyclical distribution as usual and did not show significant changes on the angiogenesis. It seems that ERs do not play a role in regulation of uNK cells [24]. A recent study discovered a new orphan receptor- estrogen receptor related beta (ERRβ, ESRRB/NR3B2) located in the nuclei of uNK cells. The receptor shares significant sequence homology with ERα and β and might transduce the effects of estrogen [25]. This provides an alternative evidence that classical mechanism is still applicable in uNK cells, yet not through conventional ERs. While we continue to identify novel subpopulation of ERs in the nuclei of uNK cells, conventional ERs, ERα and ERβ have been trafficked into cell surface to initiate intracellular signaling pathway in mice hypothalamus and striatal neurons. Especially ERβs were found to couple to metabotropic glutamate receptor 2 (mGluR2) on cell membrane and activate its intracellular signaling to inhibit phosphorylation of transcription factor cAMP-response element binding protein (CREB) in the cells (Fig. 1) [26]. However, the presence of non-classical pathways in uNK cells is unknown.

As described above, PRs have not been identified in uNK cells. Effects of progesterone are postulated to perform by other common nuclear receptor-glucocorticoid receptors (GR), because progesterone was illustrated to share structural similarity with glucocorticoid [23]. Progesterone was proved to inhibit CD69 and IFN-γ expression of human uNK cells and this effect cannot be reversed by CDB-2914, an antibody specific to progesterone. However, RU486 (antagonist of progesterone and glucocorticoid) could restore expression of CD69 and IFN-γ on uNK cells, which indirectly proved GR may be the target of progesterone [19]. Except for classical nuclear receptor, non-classical pathways of progesterone may also play an indispensable role in such regulation. In fact, the non-classical pathway of progesterone has been studied by many investigators over the past 20 years. Membrane receptor component 1 (PGMRC1) and progesterone membrane receptors (mPRs), two membrane proteins unrelated to classical PRs (Fig. 1), have been proven to be employed in the brain and reproductive tissues in mammals [26–28]. In human sperm and female reproductive tissue, they function as G protein- coupled receptors (GPCRs) to activate downstream activated or inhibitory G-proteins [29]. What’s more, in human NK cells, depolarization induced by steroid-like Na^+ ^channel (SLC) was identified due to progesterone [30]. Whether uNK cells employ the same mechanism as the cells, additional researches are still required.

In addition to direct effects of sexual hormones on the uNK cells, hormones could also regulate uNK cells indirectly, via action on neighboring cells that serve as intermediaries. In vitro studies indicate that estradiol and progesterone can reduce IL-18 level in the cultured endometrial stromal cells from patients who experienced spontaneous abortion, which helps to improve the pregnant outcomes [31].This is because IL-18 is a strong enhancer of IL-12 that can behave as proinflammatory cytokine engaging in cytolytic effects of uNK cells at excessive doses to cause pregnancy loss (Fig. 1) [32]. Otherwise, IL-18 synthesis were also found in monocyte-derived dendritic cells (mDCs) instead of stromal cells, thus, mDCs are also regarded as a potential intermediators of sex steroid hormones on uNK cells [33].

Collectively, there is some evidence related to potential mechanisms involved in the regulation of progesterone and estrogen on uNK cells. It may act through classical steroid receptors in the nuclei, non-classical pathways or through intermediary cells in the surrounding of uNK cells. Among them, non-classical pathways referred to intracellular kinase signalings or and membrane-bound receptors are mainly responsible for rapid effects of the hormones, which can be considered as a possible mechanism of dramatic tissue remodeling occurring in the early pregnancy. Thus, non-classical regulation of estrogen and progesterone on uNK cells is a proposed subject for future research.

### Regulation of conceptus on the uterine NK cells

Except for ovaries, conceptus also plays a vital role in the regulation of uNK cells. However, the regulations were less discussed in previous review literatures. In fact, conceptus can signal to uNK cells in more various ways than ovaries, which mainly rely on endocrine signaling (e.g. estrogen, progesterone). Conceptus nearly utilizes all possible signaling ways to deliver signals to uNK cells. These signals include: (1) Human chorionic gonadotropin (hCG), endocrine molecules act on the whole body via entering blood stream; (2) cytokines and chemokines, paracrine signals act locally via simple diffusion; (3) HLAs, contact-dependent signals act directly by binding cell surface receptors on target cells.

### Hormonal regulation: hCG

HCG is a glycoprotein hormone exclusively secreted by trophoblast cells during pregnancy [34]. It can be detected in the blood on 10 days after fertilization and peaks at 10th and 11th week of pregnancy, and then later declines since 12th week [35]. This temporal distribution of hCGs implicates its roles in early pregnancy and coincides with the life cycle of uNK cells. Therefore, hCG can be considered as a potential regulator of uNK cells. Although hCG has been ever added into endometrial leukocyte-rich fractions in culture medium, it made no significant changes on numbers of CD56bright (uNK) cells in the experiment [6]. However, the role of hCG in regulation of uNK cell proliferation was re-examined in a recent study. The research observed that mature hCG molecules with N-linked carbohydrate side chains could promote uNK cell proliferation. But the impact is not achieved through classical hCG/LH receptors for hCGs, it acts via mannose receptors (CD206). Only hCG molecules with carbohydrate chains can bind to the carbohydrate receptors and exert their actions on uNK cells [36]. The divergence in the above experiments may be attributed to the different types of hCG molecule used. The hCG molecules added in the previous experiment was believed to be de-glycosylated and cannot be recognized by the mannose receptors (CD206) on uNK cells. Another probable cause of failure in previous experiment is the culture medium contained too excess D-mannose, which was originally used to support cell growth. D-mannose molecules serve as competitive antagonists binding to the active sites of CD206 receptors and impair access of glycosylated hCG molecules to the receptor binding sites, resulting in an unsuccessful activation of uNK proliferation. Evidence above shed new insight into cross-talk between hCGs and uNK cells. Still, additional interactions are remained to be established.

### A complicated network of regulation: Cytokine

Prior work has illustrated the regulation of endometrium-derived cytokines on conceptus [37–39]. As several cytokines (IL-1, IL-6, IL-10, TNF-α, TNF-β) secreted by conceptus have been found in the maternal-fetal interface [37], studies related to reversed regulation of conceptus on the endometrium are onset. uNK cells, as the predominant population of lymphocytes in the endometrium, are the primary targets for conceptus-derived cytokines and relevant evidence of regulation has been ascertained progressively using clinical samples and rodent models.

Prolactin-like protein A (PLP-A) and prolactin family 8 subfamily a member 2 (PRL8A2) are two well-studied members of prolactin family in this field. They are a subset of conceptus-derived cytokines with unique functional characteristics. They neither utilize classical receptors for prolactin nor increase production of IFN-γ in uNK cells [40, 41]. In fact, the ability of uNK cell to secret IFN-γ is downregulated by the two prolactin-like proteins. How the proteins achieve their biological activities, mechanisms involved are still unclear, only intracellular Ca2+ mobilization was detected upon activation of these molecules [40, 42]. It is noteworthy that, through respective receptors, the prolactin-like proteins and prolactin can contribute to regulation of uNK cell simultaneously without disturbance to each other. In this way, PLP-A and PRL8A2 can modulate effects of prolactin to avoid inappropriate release of IFN-γ in uNK cells.

Chemokines constitute a group of cytokines that control communication and migration of immune cells. According to location of their cysteine residues, chemokines are classified into four groups: CXC, CC, CX3C and XC [43]. CXC chemokines are the first family to describe, which are involved in the regulation of conceptus on uNK cells. Through comparing chemokine receptor repertoires of cNK and uNK cells, preferential expression of CXCR3 and CXCR4 has been found on CD16- uNK cells. Then CXCL12, a ligand to CXCR4, was demonstrated to be broadly expressed in invasive trophoblast cells in vivo [44]. In vitro experiment was also performed, culture of trophoblast cells showed concentration of CXCL12 can be up to 384.6 ± 90.7 pg/ml after 60 h incubation, whereas ligands for CXCR3 were all below minimal detectable concentration after 48 h incubation [44, 45]. Based on data above, we would predict that, for conceptus, CXCL12/CXCR4 axis is the main signaling of CXC family to induce recruitment of uNK cells. CXCR7 is another receptor for CXCL12 and has ~10 folds higher affinity to CXCL12 in comparison to CXCR4 [46]. Researchers postulated CXCL7 might have an overriding advantage in mediating CXCL12 signaling. However, both immunohistochemistry and flow cytometry failed to identify CXCR7 molecules on peripheral NK cells of mice and human [47]. Thus, CXCR7 seemed not to be another desirable candidate receptor of CXC family in such recruitment. Interestingly, CXCL12/CXCR4 axis was also found to participate in cNK differentiation in the uterus through JNK1/2/MAPK and ERK/MAPK signaling pathways [48]. CXCL-12 molecules stimulated uNK cells to generate diverse factors to support conceptus invasion, which further complicated functions of CXCL12-mediating signaling pathways.

Both human and rodent studies have also suggested a potent role of monocyte inflammatory protein (MIP)-1α and macrophage chemotactic protein (MCP)-1 in migration of peripheral NK cells [49–51], which belong to CC chemokine family. Peripheral NK cells of human and mice constitutively express CCR2 for MCP-1, as well as CCR5 for MIP-1α in response to their chemotactic regulation [52]. Because MIP-1α and MCP-1 are also present in uterus during estrous cycle and increase in the early pregnancy, they are likely to be involved in the recruitment of uNK cells. In human model, Penelope M. Drake et al. confirmed localization of MIP-1α mRNA and protein in cytotrophoblast cells. At the same time, subsequent addition of anti-MIP-1α antibodies eliminated chemotaxis of 66.8 ± 20.0% CD56bright NK cells in cytotrophoblast conditioned medium, which implied that MIP-1α is responsible for conceptus-mediated chemotaxis of CD56bright NK cells in human [53]. As for MCP-1, although its level is in parallel to number of uNK cells in human endometrium, its expression in conceptus has not been proved. In mouse model, Chantakru et al. suggested recruitment of NK cells in the uterus of pregnant mice is independent of chemotaxis of MIP-1α and MCP-1, whether diameter or distribution of uNK cells was all identical in CCR2, CCR5 and MIP-1α knock-out mutants and controls [52]. So far, no other researches have illustrated the exact role of MIP-1α/CCR2 or MCP-1/CCR5 axis of uNK cells in mice models. Thus, chemotactic ability of conceptus-derived CC chemokines on uNK cells may be only conserved in human rather than rodents.

### Juxtacrine regulation: a novel but vital signaling specific to conceptus

One of significant actions of NK cells in the peripheral blood is to attack infected cells without prior sensitization. They can distinguish infected cells from normal cells by recognizing alternation in MHC class I molecules on cell surface [54]. In human, the MHC class I molecules that play an important role in NK cell recognition are members of MHC class Ia (HLA-C) and Ib (HLA-E, HLA-F, HLA-G, MIC) [55]. Among them, only HLA-C, HLA-E and HLA-G are particularly expressed by fetal- derived trophoblast cells, the only cells that directly contact to NK cells in the uterus [56, 57]. Thus, the molecules can serve as ligands to activated or inhibitory receptors on uNK cells to regulate their activities in a contact-dependent manner. HLA-E and HLA-G molecules constitute a more complicated network of regulation on uNK cells, which will be discussed in Chapter III. Here we focus on the interaction between HLA-C molecules and uNK cells.

**Fig. 2:**
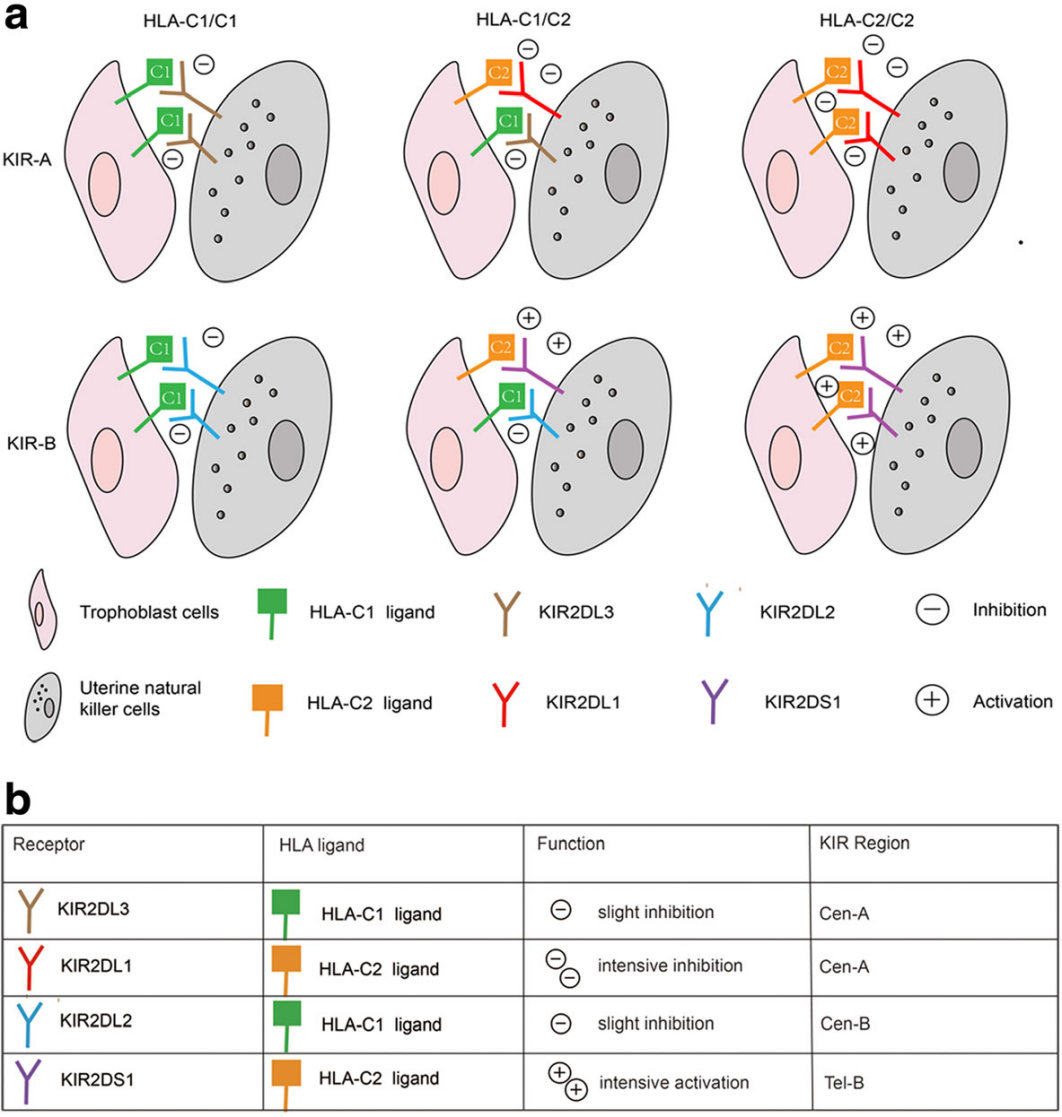
KIR-A/homologous C2 and KIR-B/homologous C1 are two high-risk combination. **a** All possible combination of maternal KIR haplotypes with trophoblastic HLA-C ligands. Combinations with intensive inhibition are failure to activate uNK cells to secrete relevant angiogenic cytokines and more vulnerable to undergo pregnancy losses. Thus, KIR-A/homologous C2 and KIR-B/homologous C1 are two high-risk combinations than other combinations. **b** Binding properties of the main KIRs for fetal HLA-C

According to a dimorphism at position 80 of the α1 domain, HLA-C molecules on trophoblast cells are classified into 2 classes (Fig. 2): C1 and C2 allotype. C1 allotype are mainly bound by inhibitory KIR2DL2 and KIR2DL3 on the uNK cells, whereas C2 allotype are preferentially bound by inhibitory KIR2DL1 and activated KIR2DS1 [58]. Killer immunoglobulin-like receptors (KIRs) are molecules developed by NK cells in recognition of HLA-C molecules on cell surface and divided into activated and inhibitory receptors based on their action on NK cells [59]. Dependent on presence of activated receptors, over 350 KIR genotypes are basically divided into 2 haplotypes: A (only inhibitory receptors) and B (inhibitory and activated receptors), which can be subdivided into centromeric and telomeric regions (Cen-A, Tel-A, Cen-B, Tel-B) [60, 61]. The great diversity of maternal KIRs and fetal HLA-C ligands means that a huge number of combinations will be generated in pregnancies and combinations failed in activation of uNK cells are attributed to pathogenesis of preeclampsia, fetal growth restriction and recurrent pregnancy, which can be explained by poor angiogenesis [62]. Some studies have demonstrated frequencies of affected pregnancies increased in KIR AA mother, especially accompanied with homologous C2 fetus [63]. Recently, another high-risk combination is identified by direct embryo HLA-C genotyping, which is the haplotype B/homologous C1. In overall, the two combinations can cause a 51% increased risk of pregnancy loss over all other combinations. What’s more, the interaction of HLA-C and KIRs not only can determine the possibility of pregnancy losses but also the timing of them. Because the homologous C1 embryos were observed to undergo more biochemical pregnancy losses than clinical losses, which caused its latent discovery in clinic practice compared to the haplotype A/homologous C2 combination [64].

### Combined action of ovary and conceptus on uNK cells

Evidence above has shown that whether ovary or conceptus plays a vital role in the regulation of uNK cell activities, however, it is also important to note that they can act in synergistical manners. Animal and human studies have suggested that progesterone regulates expression of progesterone induced blocking factor (PIBF) and HLA-G in trophoblast cells to modify uNK cells function. Initially, PIBF proteins were found to be synthesized by γδ T lymphocytes in response to progesterone in circulatory system. The proteins exert their immunosuppressive effects on peripheral NK cells and maintain normal pregnancy [65]. Then PIBF proteins attract considerable attention as a novel part of non-classic regulatory pathway of progesterone. In fact, γδ T lymphocytes are not the only cellular resource of them. A human study illustrated that trophoblast cells in the placenta could express PIBF proteins of 30, 50 and 90 kDa in first trimester [66]. Because neither PRs nor homologies were verified in uNK cells [23], such non-classical regulation was required by uNK cells in response to progesterone. Moreover, as an immunosuppressant, PIBF proteins are likely candidates in mediating immune tolerance of uNK cells to conceptus. The postulation has not been confirmed until Bogdan et al. demonstrated PIBF proteins co-localized with perforins in DBA^+^ uNK cells [21]. In the study, progesterone was administrated to these cells and reduced 47% perforin^ +^ cells from them, which directly proved the presence of the non-classical pathway of progesterone in the regulation of uNK cells.

In studies of HLA-G molecules, progesterone is also shown to be correlated with expression of HLA-G molecules on cell surfaces. Progesterone upregulates HLA-G molecules via a novel progesterone response element (PRE), which is located on promoter of HLA-G genes in JEG-3 cell models and shares to 60% similarity to mouse mammary tumor virus PRE [67]. Within the element, it can enhance HLA-G expression on JEG-3 carcinoma cells and human cytotrophoblast cells in vitro [68]. What’s more, normal human endothelial cells of heart vessel and smooth muscle cells do not present HLA-G molecules on their surface or in the cytoplasm, but they are able to present the molecules after progesterone treatment [69]. Thus, HLA-G is likely to be another intermediary molecule involved in progesterone-mediated indirect modulation of uNK cells.

Unlike classical MHC class Ιa molecules, HLA-G molecules are exclusively expressed by fetus-derived cells like extravillous cytotrophoblast cells or chorionic endothelial cells in the maternal-fetal interface [9, 70]. In addition, their presence in endometrial stroma cells, which are maternal-derived, has also been proved [71]. However, not all HLA-G molecules are expressed on cell surface. Some alternative splice transcripts of HLA-G mRNA lack exons encoding transmembrane or cytoplasmic translated domains and are translated as soluble forms [72]. For NK cells, they only have 2 types of receptors for HLA-G molecules: one is ILT2 for membrane-bound isoforms; the other is KIR2DL4 for soluble isoforms [73, 74]. Despite of different isoforms, engagement of HLA-G molecules yields completely the same consequence that HLA-G molecules inhibit cytotoxicity of uNK cells and increase the secretion of IL-6, IL-8, and TNF-α, which play roles in spiral artery modification [75]. HLA-G molecules also can reduce cytotoxicity of uNK cells without the help of their cell surface receptors. A leader peptide derived from HLA-G molecules can bind to HLA-E molecules on cell surface and stabilize their expression. The corresponding receptor of HLA-E that is highly expressed on uNK cells is NKG-2A. NKG-2A is an inhibitory receptor and present on uNK cells in a complex with CD94 [76]. Thus, HLA-G molecules can indirectly activate inhibitory NKG-2A/CD94 to reduce uNK cell cytotoxicity by promoting HLA-E molecules expression.

## Conclusion

**Fig. 3:**
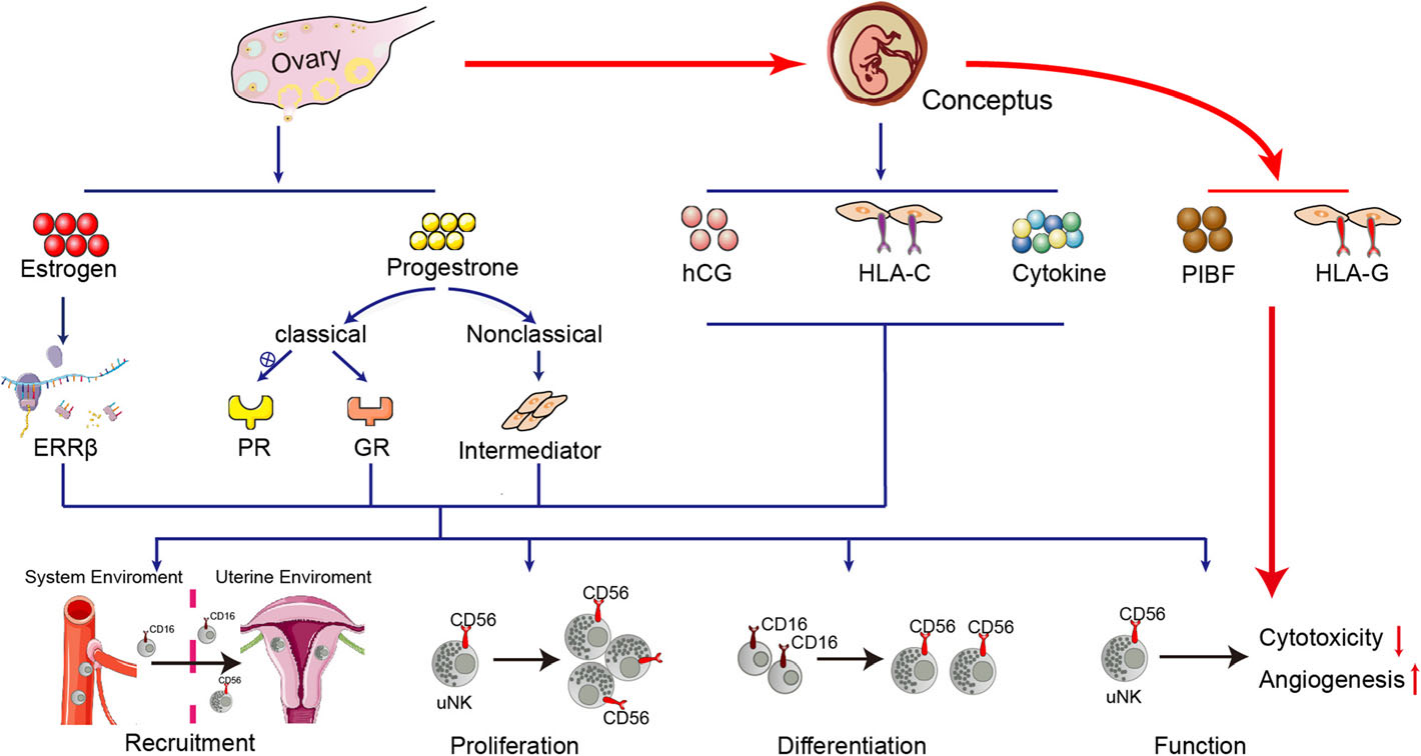
The regulation of ovary and conceptus on the uNK cells. In early pregnancy, endocrine signaling (e.g. estrogen and progesterone) of ovarian cells can regulate recruitment, proliferation, differentiation and function of uNK cells via direct action on intracellular nuclear receptors (e.g.ERRβ, GR) or through intermediary cells in the surroundings (e.g. T lymphocytes and endometrial stromal cells). Compared to ovaries, besides endocrine signaling (e.g. hCG), conceptus also utilizes juxtacrine signaling (e.g. HLA-C) and paracrine signaling (e.g. cytokines) to facilitate activities above of uNK cells. In addition, under the influence of ovarian hormones, conceptus can increase expression of PIBF and HLA-G molecules to reduce cytotoxicity of uNK cells and promote angiogenesis

In general, ovary and conceptus can regulate activity of uNK cells in a separated or combined manner (Fig. 3). Dysregulation of uNK cells has been linked to gestational implications such as recurrent pregnancy loss, preeclampsia and fatal growth retardation. Given poor understanding of the mechanisms involved in the pathogenesis of the diseases, current therapies are all specialized for the hormonal dysregulation of uNK cells. Through giving a more comprehensive overview of regulatory network of them in the uterine microenvironment, our work can hopefully provide more conceptus-derived targets for the development of novel and effective therapies.

## Abbreviations

cNK: Circulatory natural killer
CREB: cAMP- response element binding protein
ERRβ: Estrogen receptor related beta
FSH: Follicular stimulating hormone
GPCR: G-protein coupled receptor
GR: Glucocorticoid receptor
HCG: Human chorionic gonadotropin
HLA: Human leucocyte antigen
KIR: Killer immunoglobulin-like receptor
LH: Luteinizing hormone
MCP: Macrophage chemotactic protein
mDCs: Monocyte-derived dendritic cells
mGluR2: Metabotropic glutamate receptor 2
MHC: Major histocompability complex
MIP: Monocyte inflammatory protein
mPR: Progesterone membrane receptor
PGMRC1: Progesterone membrane receptor component 1
PIBF: Progesterone induced blocking factor
PLP-A: Prolactin-like protein A
PRE: Progesterone response element
PRL8A2: Prolactin family 8 subfamily a member 2
SLC: Steroid-like Na + channel
uNK: Uterine natural killer
